# Institutional Outdoor Work and Administration

**Published:** 1911-10-28

**Authors:** 


### INSTITUTIONAL OUTDOOR WORK AND ADMINISTRATION.

### V.—Grounds and Land.

It often happens that a new institution has to be opened before there is time to lay out the grounds at all fully. This is, however, in reality rather an advantage, since it thus becomes possible to do things much more economically. Nurserymen have a way of cramming the area for planting as full as it will hold of expensive shrubs that after a year or two are fighting for air space and killing each other out. And even the most modest-sized lawn costs a considerable amount of money if turfs have to be bought and carted. Sowing grass, on the other hand, is an operation within the capacity of anyone, and the well-known seedsmen, Messrs. Carter, have a useful little green book which gives the plainest directions. The two suitable seasons for sowing are the end of February and the end of August, the latter for preference. Dig plenty of stable manure into the surface, and in dry weather roll and cross-roll until firm enough not to show a foot-mark. Then lightly rake, sow with a free hand, lightly rake again, and finally roll. So soon as the young blades are an inch high, cut by hand and keep on cutting—this is the only troublesome part of the business—and the result as regards fresh green colour and evenness of surface will be much superior to a turf-laid lawn.

What one wants in the small shrubbery beds surrounding buildings are plants that are quick growers, cheap, hardy, easy to grow, and evergreen —or at least ornamental all the year round. The following species are given as combining these recommendations: Scotch fir, cotoneaster, evergreen privet, cupressus Lawsoniana, and the hardier laurels. In sunny spots common broom-seed sown in April will make in the second year good bushes of a graceful appearance over four feet high. Juniper and English yew form a good contrast, but are slower-growing than those already given and more expensive. The advantage of native varieties is that they may be relied upon to stand any winter. They are also less exigent in the matter of soil. If there be any thought of making a small timber plantation, then as a general rule larch is the kind to plant. All shrubs and young trees, it should be remembered, have a much better chance if put in in the autumn—when the earth is warm—and if the ground between them is kept clear of undergrowth of any kind. Firm staking is an essential, wire being used and the bark shielded with sacking.

The permanent roads about an institution, well "pitched" (or "penned") with stone, covered with mud or tar-bound road metal, will of course be found ready made. But occasion generally arises for additional and rather less solidly constructed ways to lead to various points. It is of much advantage to use ashes for these, since ashes are readily procurable from the engine-house, and, indeed, soon accumulate in an unsightly heap if not disposed of in some way. The first step, then, is to dig down to firm subsoil. The top layer thus removed is of value for forming beds or for increasing the depth of soil in neighbouring cultivable areas. Thus in a garden it is well worth while to spend trouble in this way in making paths, as a deep soil is obtained, with consequent lessening of exhaustibility, and the paths are firmer and freer from weeds. After excavation, a foundation must be laid of stones just small enough to pass a four-inch ring, eked out with old tins, broken jam-jars and the like rubbish. Then cover with plenty of ashes and roll and cross-roll. If a line of field drain should cross the track, remove the earth covering it and fill in rather loosely with large clinker before putting on the stones and ashes. This will drain the bed of the road; but if ashes are put directly into contact with the pipes the water cannot percolate. On the high side of the road a surface channel must be provided, to prevent heavy rain or snow washing the surface away. Hints as to excavation and removal of earth on a larger scale and construction of small embankments, or "batters" as navvies call them, can be got by watching a gang of these knights of the pick at work. Their technique is elementary, but necessary to good results.

The utilisation of any land attached to the institution will depend much upon particular circumstances, as, for instance, whether horses are kept or not. If they are, obviously it will be worth while to grow oats and hay for their keep, as most horse-owners in the country do. Horses, by supplying carting needs and furnishing the best manure, fit so well into a general scheme of development of even a small estate that this question will now be considered. It certainly does not pay, however, to keep them solely for leading coal. The economical plan is to get two "half-breds"—clean-legged carthorses which can draw a load of 12 cwt., and also trot fast enough to do the station work with spring cart or waggonette. Indeed, practical people prefer animals lighter than a regular cart-horse for leading coal, as being quicker on the road and eating less.

Oats, like potatoes, are a good first crop on land, and have moreover the advantage of furnishing food material in the straw, which may be chopped for cattle or pigs. The proprietors of the threshing machines which travel the district will usually come three or four times a year if necessary. Plough lime into the rough land in autumn, clean with a harrow, and the winter's frost will do the rest in triturating the surface. Manure will be needed, of course, as always. If the advantageous plan be followed of sowing clover among the young oats, then after the first reaping there is left on the ground a fairly established crop, which in the two ensuing seasons makes the new land hay which horses need. After this it can be left as a permanent pasture, or be ploughed up.

* Former articles of this series appeared in The Hospital of August 26, September 9, and September 23.

